# Supplementary material for: FastqPuri: high-performance preprocessing of RNA-seq data
Source: BMC Bioinformatics. 2019 May 3;20:226. doi: 10.1186/s12859-019-2799-0 (PMC6500068; doi:10.1186/s12859-019-2799-0)
Supplement: Supplementary file 2 — Archive of FastqPuri. Archive containing all files needed to install and run FastqPuri v1.0.6. Date stamp March 22, 2019. (GZ 47,819 kb) [file 12859_2019_2799_MOESM2_ESM.gz › FastqPuri-1.0.6/html/init__makeTree_8h_source.html]

FastqPuri: include/init\_makeTree.h Source File


FastqPuri


- include

init\_makeTree.h

Go to the documentation of this file.

1 /\*\*\*\*\*\*\*\*\*\*\*\*\*\*\*\*\*\*\*\*\*\*\*\*\*\*\*\*\*\*\*\*\*\*\*\*\*\*\*\*\*\*\*\*\*\*\*\*\*\*\*\*\*\*\*\*\*\*\*\*\*\*\*\*\*\*\*\*\*\*\*\*\*\*\*\*

2  \* Copyright (C) 2017 by Paula Perez Rubio \*

3  \* \*

4  \* This file is part of FastqPuri. \*

5  \* \*

6  \* FastqPuri is free software: you can redistribute it and/or modify \*

7  \* it under the terms of the GNU General Public License as \*

8  \* published by the Free Software Foundation, either version 3 of the \*

9  \* License, or (at your option) any later version. \*

10  \* \*

11  \* FastqPuri is distributed in the hope that it will be useful, \*

12  \* but WITHOUT ANY WARRANTY; without even the implied warranty of \*

13  \* MERCHANTABILITY or FITNESS FOR A PARTICULAR PURPOSE. See the \*

14  \* GNU General Public License for more details. \*

15  \* \*

16  \* You should have received a copy of the GNU General Public License \*

17  \* along with FastqPuri. \*

18  \* If not, see <http://www.gnu.org/licenses/>. \*

19  \*\*\*\*\*\*\*\*\*\*\*\*\*\*\*\*\*\*\*\*\*\*\*\*\*\*\*\*\*\*\*\*\*\*\*\*\*\*\*\*\*\*\*\*\*\*\*\*\*\*\*\*\*\*\*\*\*\*\*\*\*\*\*\*\*\*\*\*\*\*\*\*\*\*\*\*/

20

29 #ifndef INIT\_MAKETREE\_H\_

30 #define INIT\_MAKETREE\_H\_

31

32 #include "defines.h"

33

37 typedef struct \_iparam\_makeTree {

38  char \*inputfasta;

39  char outputfile[MAX\_FILENAME];

40  int L;

41 } Iparam\_makeTree;

42

43 void printHelpDialog\_makeTree();

44

45 void getarg\_makeTree(int argc, char \*\*argv);

46

47 #endif // endif INIT\_MAKETREE\_H\_

\_iparam\_makeTree::L

int L

**Definition:** init\_makeTree.h:40

getarg\_makeTree

void getarg\_makeTree(int argc, char \*\*argv)

Reads in the arguments passed through the command line to makeTree. and stores them in the global var...

**Definition:** init\_makeTree.c:64

\_iparam\_makeTree::inputfasta

char \* inputfasta

**Definition:** init\_makeTree.h:38

Iparam\_makeTree

struct \_iparam\_makeTree Iparam\_makeTree

contains makeTree input parameters

MAX\_FILENAME

#define MAX\_FILENAME

**Definition:** defines.h:37

defines.h

Macro definitions.

\_iparam\_makeTree::outputfile

char outputfile[MAX\_FILENAME]

**Definition:** init\_makeTree.h:39

printHelpDialog\_makeTree

void printHelpDialog\_makeTree()

Function that prints makeTree help dialog when called.

**Definition:** init\_makeTree.c:43

\_iparam\_makeTree

contains makeTree input parameters

**Definition:** init\_makeTree.h:37


---

Generated on Mon Mar 19 2018 23:42:01 for FastqPuri by  

 1.8.14
